# Local suffering and the global discourse of mental health and human rights: An ethnographic study of responses to mental illness in rural Ghana

**DOI:** 10.1186/1744-8603-5-13

**Published:** 2009-10-14

**Authors:** Ursula M Read, Edward Adiibokah, Solomon Nyame

**Affiliations:** 1 Department of Anthropology, University College London, UK; 2 Kintampo Health Research Centre, Kintampo, Brong Ahafo, Ghana

## Abstract

**Background:**

The *Global Movement for Mental Health *has brought renewed attention to the neglect of people with mental illness within health policy worldwide. The maltreatment of the mentally ill in many low-income countries is widely reported within psychiatric hospitals, informal healing centres, and family homes. International agencies have called for the development of legislation and policy to address these abuses. However such initiatives exemplify a top-down approach to promoting human rights which historically has had limited impact at the level of those living with mental illness and their families.

**Methods:**

This research forms part of a longitudinal anthropological study of people with severe mental illness in rural Ghana. Visits were made to over 40 households with a family member with mental illness, as well as churches, shrines, hospitals and clinics. Ethnographic methods included observation, conversation, semi-structured interviews and focus group discussions with people with mental illness, carers, healers, health workers and community members.

**Results:**

Chaining and beating of the mentally ill was found to be commonplace in homes and treatment centres in the communities studied, as well as with-holding of food ('fasting'). However responses to mental illness were embedded within spiritual and moral perspectives and such treatment provoked little sanction at the local level. Families struggled to provide care for severely mentally ill relatives with very little support from formal health services. Psychiatric services were difficult to access, particularly in rural communities, and also seen to have limitations in their effectiveness. Traditional and faith healers remained highly popular despite the routine maltreatment of the mentally ill in their facilities.

**Conclusion:**

Efforts to promote the human rights of those with mental illness must engage with the experiences of mental illness within communities affected in order to grasp how these may underpin the use of practices such as mechanical restraint. Interventions which operate at the local level with those living with mental illness within rural communities, as well as family members and healers, may have greater potential to effect change in the treatment of the mentally ill than legislation or investment in services alone.

## Background

The contribution of mental disorders to the burden of chronic disease has been re-affirmed in the latest update to the Global Burden of Disease (GBD) study. This identifies neuropsychiatric conditions including depression, psychoses and alcohol use disorders, as the leading causes of disability worldwide, representing a third of all years of healthy life lost to disability among adults [1]. According to this study, the burden of disability is highest in African countries, presumably due to the impact of poverty and low levels of treatment and rehabilitation for chronic diseases. Within sub-Saharan Africa the majority of those with mental disorders receive no treatment from mental health services. In a study in Nigeria, for example, only 9% of 1,682 people diagnosed with anxiety, mood or substance use disorder had received any treatment, and even this treatment was judged to be inadequate [2]. Mental health care is underfunded across the continent compared to other health concerns. According to the World Health Organization (WHO), 70% of African countries spend less than 1% of their health budgets on mental health [3]. Even then the majority of government funding for mental health is consumed in maintaining large psychiatric institutions, with very little allocated for the treatment and prevention of mental disorders in the community. In common with general health care and other public services, psychiatric services tend to be concentrated within the urban centres of most countries of sub-Saharan Africa. This means that the poorest members of these countries who live in rural areas far from the capitals and major cities face the greatest challenges in accessing mental health care.

In response to these deficits in mental healthcare, 2008 witnessed the launch of *The Global Movement for Mental Health *. The movement has three key objectives: the scaling up of mental health services, protecting human rights, and promoting research in low- and middle-income countries. This movement is the latest development in a global push for improved mental health care which began in 2001 with the World Health Report on mental health [4]. It received renewed impetus in 2007 with the publication of the *Lancet *series on mental health which highlighted the paucity of attention to mental health in the global public health forum culminating in a 'call for action' [5]. This call, which forms the foundation of the *Global Movement for Mental Health*, suggests that Government ministries should 'identify and scale up a priority package of service interventions or components that can form the backbone of a national mental health system that provides effective interventions and human-rights protection' [5]. Recommended strategies are in line with long-standing recommendations for the delivery of mental health care which emphasize the need for decentralisation, community-based mental health care, and the integration of mental health within primary care. The movement also suggests that governments of low- and middle-income countries should establish a national body to monitor and protect the human rights of people with mental disorders, and 'promote adoption and implementation of national mental health legislation in accordance with international human-rights instruments' [5]. However this focus on state interventions to promote human rights faces additional challenges in countries with emerging economies, and weak systems of governance and civil participation. Many governments of sub-Saharan Africa for example, have historically shown little respect for the human rights of their populations, whether mentally ill or otherwise. This paper considers the challenges facing the protection of the human rights of people with mental illness drawing on the results of ethnographic research in Kintampo, a rural community in Ghana, West Africa.

### Mental health and human rights

Reports by NGOs and the media regarding the widespread maltreatment of the mentally in low-income countries of sub-Saharan Africa, including graphic images of people in chains, have provoked shock and outrage amongst many observers, and led to urgent calls for reform. It is striking that such appeals have generally come not from the communities affected, but from concerned visitors and experts from the international scene - NGOs, WHO and internationally prominent psychiatrists. These concerns are far from new. In 1991 the UN adopted the 'Principles for the Protection of Persons with Mental Illness and for the Improvement for Mental Health Care' (commonly known as the MI Principles) [6]. In response to the lack of progress in meeting the minimum standards enshrined in these principles, the Institute of Psychiatry in the UK launched 'Principles to Respect', an 'Initiative on Mental Health and Human Rights' which aimed to promote the MI principles within psychiatric facilities worldwide [7]. Most recently the UN Convention on the Rights of Persons with Disabilities (including within this definition those with 'mental impairments') was passed in 2006, although it remains to be ratified by many countries [8].

All these initiatives draw on the principles of human rights to prohibit the unlawful deprivation of liberty and the use of 'cruel, inhuman or degrading treatment or punishment'. Countries are supported by international agencies such as WHO to develop mental health legislation and policy as a step to improved services and the outlawing of human rights abuses [9]. However, despite the best intentions of these initiatives, such 'top-down' approaches are in danger of failing to bring about change in the communities most affected, as evidenced by the persistence of human rights abuses in states which have been signatory to international treaties and conventions, as well as reproducing human rights rhetoric within their national constitutions and legislation. As observed elsewhere, a proliferation of human rights documents has not correlated with a decrease in human rights abuses [10]. The stark fact remains that in spite of decades of international human rights initiatives, throughout many countries of sub-Saharan Africa the chaining and other maltreatment of the mentally ill remains routine.

It is perhaps unsurprising that human rights abuses are often linked to poor standards of mental health care in low-income countries and the need for methods of restraint in the absence of easily available neuroleptics. Data produced by WHO such as the Mental Health Atlas [3], in which the scarcity of psychiatric resources such as hospital beds, psychiatrists and mental health spending in sub-Saharan Africa is all too clear, would seem to support this conclusion. However such data fails to enumerate the contributions of family members and religious healers, as well as other informal resources, which form the backbone of care for the mentally ill in many countries of sub-Saharan Africa. In addition, attitudes to the care of those with mental illness seem to vary even between countries with similar levels of economic development. Whilst chaining of the mentally ill is commonplace in countries of sub-Saharan Africa, in Peru this does not occur, even in remote rural communities where psychiatric services are scarce (David Orr, University College London, personal communication). This suggests that responses to the mental illness of a family member are influenced by social norms regarding the control of mental illness which are in turn informed by historical, cultural and symbolic practices. Such social norms become the accepted, even expected, practices in response to mental illness, and hence may not evoke widespread protest, particularly at the community level.

This paper draws on anthropological research with people with mental illness, their families and healing practitioners within rural communities in Ghana, to gain an understanding of how practices such as the chaining and beating of those with mental illness are embedded within sociocultural meanings and responses evoked by madness or mental illness. An ethnographic approach involving long-term research within the field permits one to trace the trajectory of family responses to mental illness in which chaining often forms part of a long period of help-seeking. This research enabled encounters with families before, during and after the use of chains, and was thus able to track changes in family responses over time.

### Mental health policy and service delivery in Ghana

Like many countries of sub-Saharan Africa, Ghana's psychiatric services have their origins in the colonial period with the establishment of an asylum in the capital, Accra. This was largely custodial rather than therapeutic in function and served to detain those with mental illness who had increasingly come to the notice of the colonial authorities, particularly in urban areas [11]. Two further psychiatric hospitals were established following independence offering inpatient and outpatient treatment for mental disorders. All three hospitals are located in the south of the country and from their inception have suffered from overcrowding and understaffing leading to poor quality of care. Despite several initiatives to improve mental health services, including the training of community psychiatric nurses and the opening of regional psychiatric units, the vision of a comprehensive community mental health system held by the first African psychiatrist in the country, E.M. Forster [12], has yet to be fulfilled. Political apathy towards mental health, combined with widespread stigma, hamper the progress of mental health care in the country. Traditional healers, and increasingly pastors of the Pentecostal churches, continue to deal with the greatest proportion of those with mental disorders. Whilst these often address the spiritual concerns of Ghanaians who use their services, there are reports of maltreatment and human rights abuse including chaining, enforced fasting, and beatings [13].

However there are some signs of a renewed impetus for mental health care within Ghana. A new mental health bill has been highly praised for its focus on human rights and community-based services [14]. The current health sector five year Programme of Work states a commitment to promoting mental health [15]. In addition to such policy initiatives, there are increasing numbers of NGOs working in mental health, and a large research programme consortium, the Mental Health and Poverty Project (MHaPP) is conducting research on mental health and poverty within four African countries including Ghana [16]. This year also saw the relaunch of the Ghana Mental Health Association, drawing together interested parties in supporting mental health in the country. In recognition of the burden of mental disorders in Ghana and the relative paucity of financial and human resources, as well as its readiness for reform, Ghana is one of the countries which has been identified by the WHO initiative *Mental Health Gap Action Programme *(mhGAP) to received intensified support to scale up treatment for mental, neurological and substance use disorders [17]. As a relatively stable democracy with a history of psychiatric innovation and a growing advocacy movement for mental health within both the health care sector and civil society, Ghana is facing a unique opportunity to pioneer improved mental health care in the West African region.

## Methods

### Fieldwork setting

The study centres around a rural town, Kintampo, in Brong Ahafo, in the central belt of Ghana. Kintampo forms a transit zone between north and south, and is home to many migrant communities now settled in the town. Kintampo also marks the boundary between two administrative districts, North and South Kintampo. The total population of these two districts is about 190,000, the majority of which live in rural areas. Some of these rural communities are strung along the main north-south road, many others are located at some distance along unpaved feeder roads. Farming is the major occupation for about seventy per cent of the population in the districts. The most widely spoken language in the district is Twi, which is spoken by the Akan, the largest ethnic group in the region as well as in Ghana as a whole, and adopted by many others as a lingua franca. Other widely used languages in the district include Hausa and English, which is the official language for government bodies such as education and health services. Over 60% of the population are Christian, nearly 30% Muslim, and around 8% follow the traditional religion, though the use of traditional shrines is more widespread than this figure would suggest.

**Figure 1:**
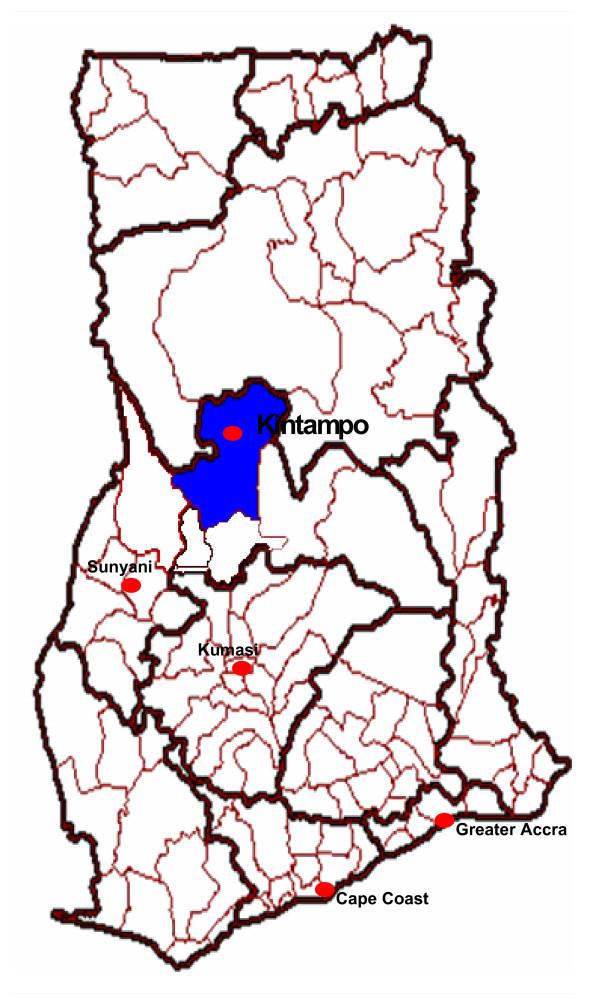
**Map of Ghana showing location of psychiatric facilities used by participants**.

There are three major sources of help for families in Kintampo North and South districts who have a relative with mental illness, including biomedical healthcare, 'traditional healing' performed by fetish priests (Twi: *akכmfoכ)*, and 'faith healing' from Christian pastors or Muslim mallams. Ghana Health Service is the main provider of biomedical care for mental illness, however treatment for mental disorders seldom penetrates to the community level. Until 2008 there were no mental health professionals throughout the two Kintampo districts. A Community Psychiatric Nurse (CPN) has now been posted to Kintampo. In theory she provides a service to the town and surrounding communities, but since she is provided with no means of transport she is limited in her capacity to conduct home visits on a regular basis, particularly to more distant settlements. Treatment for mental illness at the community level is largely through the provision of psychotropic drugs which are available from the district hospital at Kintampo and from the CPN. Clinics located in rural communities (sub-districts) are not equipped to treat mental illness. Inpatient and outpatient psychiatric care is available in Sunyani, the regional capital, where there is a psychiatric unit within the regional hospital. However the three state psychiatric hospitals provide the major source of inpatient treatment. These are all located in the south of Ghana, a day's journey from Kintampo (see figure 1).

By contrast, informal treatment providers are many and varied, their numbers easily exceeding psychiatric services. Most communities have an *כkכmfo*, a traditional healer or fetish priest, who under the instruction of the *abosom *or 'small gods', treats mental illness through the use of herbal medicines and ritual such as animal sacrifice. Also popular as sources of healing for mental illness are 'prayer camps' established by Christian pastors who provide healing through prayer, fasting and deliverance from evil spirits. One pastor in Kintampo town is well-known in the area for his power in healing those who are mentally ill, and hundreds if not thousands of pastors offer similar services throughout the country. A shrine in a small rural community in Kintampo South district is also famed for healing madness and is visited by people from as far afield as the Ashanti region and sometimes beyond. Treatment at prayer camps and shrines often involves a lengthy stay of several months; sometimes up to a year or even more. Relatives are usually expected to stay with the patient at the prayer camps and shrines to provide day-to-day care. Most frequently this is the mother, but sometimes the father, sister or another relative takes this role.

### Research design

Despite longstanding calls for the contribution of anthropology to explore the influence of culture on the experience and outcome of mental illness [18-20], there are few detailed ethnographic studies of people living with mental illness in low-income countries. Many studies provide little detail about the socio-cultural world in which people live, and the ways in which people with mental illness are treated by their families, friends or the general population [18,19,21,22]. This research draws on the methods of transcultural psychiatry which views mental illness as a function of 'the unique experience of being a member of a particular society: a society with its own characteristic web of economic constraints, social relations and beliefs' [23]. Utilising anthropological methods including participant observation, conversation and semi-structured interviews with people with mental illness, their families, healers, health professionals and community members within Kintampo town and the surrounding villages, the study aimed to discover the particularities of responses to severe mental illness as embedded within the experience of living in a rural West African community.

### Research subjects

Participants were recruited through purposive sampling at shrines, churches, prayer camps and family homes. Initially the researchers identified one shrine and two prayer camps within the Kintampo districts who frequently treated people with mental illness. The shrine regularly had 8-10 people with mental illness staying in the compound. However the two prayer camps were relatively small without a frequent turnover of patients, so a larger prayer camp was identified in Techiman, a market town thirty minutes from Kintampo, where there were greater numbers of people with mental illness. All of these healing centres took patients from across the country, though predominantly from Brong Ahafo and Ashanti regions. Permission was sought from the pastor or *כkכmfo *to approach potential participants visiting the shrine/church. Other participants were recruited from the database of an earlier epidemiological study of psychosis , patients attending the CPN clinic, and through contacts in the community (see Table 1).

**Table 1:** Sampling of cases

**Source**	**n =**
Epidemiological study of psychosis	10
Shrine	9
Prayer camp 1	3
Prayer camp 2	6
CPN	4
Word of mouth	6

TOTAL	38

The focus of the study is on those who in Twi would be described as *כbכdamfo*, or a mad person. This behaviour is identified by local informants with forms of 'wild' and anti-social behaviour and is closest to what in psychiatric terms would be labelled psychosis. Frequently described behaviours include talking to oneself, talking in a disordered way (*kasa basabasa*), acting aggressively (*gidigidi*), and dressing in dirty clothing. The study focuses on those with more longstanding forms of mental illness which involve severe disruption of perception, thought, and social functioning. The majority of those studied have been ill for periods of at least 5 years, some for much longer than this. Many traced the onset of their illness to adolescence or early adulthood.

### Fieldwork

Anthropological fieldwork requires prolonged immersion in the community under study and participation in everyday life, typically for a period of at least one year, in order for the researcher to become familiar with local practices and to minimise the reactivity of informants [24]. Fieldwork took place between October 2007 and December 2008 following a pilot study in June - July 2006. The principal researcher (UMR) lived within Kintampo during the period of fieldwork, and spent time informally with people living in the Kintampo districts, observing practices such child-rearing, food preparation, agricultural practices, social relationships and other daily routines. The fieldwork assistant (SN) was trained in ethnographic methods, including participant observation and semi-structured interviewing. He accompanied the principal researcher on visits to field sites, and provided assistance with interpretation, conducting interviews and focus groups, and arranging entry to the field. The assistant also functioned as an 'expert informant' during participant observation, to assist with the explanation of practices observed, as well as with interpretation. The research consisted of three main approaches: detailed case studies of people with mental illness, in-depth observation of treatment and healing practices for mental illness, and gathering contextual information relevant to mental illness (see Appendix 1).

Alongside interviews to elicit verbal accounts, an important part of the research involved spending time with people with mental illness and their families observing their everyday life and their integration and participation within the community, including the attitudes of others towards them. Regular visits were undertaken to the homes of families who had a relative with mental illness, to the shrine, and to the three churches treating people with mental illness. Fieldnotes were written by the researcher and the assistant to record observations and conversations following each visit.

During the course of the research over 40 homes were visited in addition to the shrine and prayer camps, and a total of 67 participants were interviewed including 25 patients, 31 carers, 3 traditional healers, 4 pastors, 1 mallam and 3 imams (see Table 2). Three interviews were in English, the rest in Twi. Wherever possible we interviewed the person with mental illness, however some were too unwell to provide consent or to participate in the interview, in which case we interviewed the main carer, usually the mother, father or sibling. In eight of the interviews the carer and the person with mental illness were interviewed together. This was due to the fact that these patients could not remember significant details of the time when they were sick, or suffered from deficits in communication or cognition which made it difficult to obtain a coherent interview alone. To obtain contextual information relevant to mental health 7 focus group discussions were held with a total of 47 participants including registered mental nurses, young people, Muslims, cannabis users, church members and parents (see Table 3). Five FGDs were conducted in Twi; two in English. Interviews were semi-structured. For those with mental illness and their family members questions focused on the history of the person's illness, the symptoms and course of the illness, possible causes, the impact of the illness on the individual and the family in terms of day-to-day life and social roles, sources of treatment employed, and the experience of such treatment, including its perceived efficacy. For healers interview questions focused on the healers' view of mental illness, including possible causes, the methods of treatment provided and the ideology/theology on which they were based, the efficacy of the treatment and the reasons for this, and views of other forms of treatment and possible collaboration or interaction.

**Table 2:** Interview participants

	**n =**
People with mental illness	25
Carers	31
Pastors	4
Traditional healers	3
Imams/mallams	4

TOTAL	67

**Table 3:** Focus group participants

	**n =**
Church members	8
Muslims (men)	7
Muslims (women)	7
Young people	8
Cannabis smokers	5
Parents	7
Registered mental nurses	5

TOTAL	47

### Data analysis

Interviews and focus groups were digitally recorded with the permission of the informants. Five assistants bi-lingual in Twi and English were recruited and trained. They transcribed the interviews and focus groups into Twi and then translated into English. All potentially identifying details were removed in the transcripts. Analysis utilised a grounded theory approach in which hypotheses were generated through close examination of the data [24]. Transcripts and fieldnotes were read and recurring themes and differences noted. The multiple methods used allowed for some triangulation of the data.

### Ethics

Ethical approval for the study was granted by University College London and Kintampo Health Research Centre (KHRC). On introduction all participants in interviews and focus groups were provided with a written information sheet and consent form which was translated into Twi. As many participants were unable to read Twi the forms were read to the participants and a verbal explanation of the research aims and methods provided. Questions were invited from participants. Participants were asked to sign consent forms, or if illiterate to provide thumb prints in the presence of a witness. Where possible the researchers aimed to interview the person with mental illness and the main carer. However if the person with mental illness was considered too unwell to provide informed consent, he or she was not interviewed.

It is not feasible nor appropriate to obtain written consent from all persons who may be involved in observation, for example a church congregation. The researcher sought the permission of those in authority at proposed sites, such as the pastor or traditional healer, before commencing observation and participation, and ensured that all persons who were involved in periods of observation were informed of the nature of the research.

Of particular concern in this study were occasions when the researchers encountered people who were being treated within the shrine and prayer camps and presented with severe and distressing symptoms. Where it was judged by the principal researcher (who has several years experience as a clinician in mental health services in the UK) that the person may benefit from psychiatric treatment, the researchers advised the person and their family of the availability of medical treatment for such illnesses and the potential benefits. Assistance was provided to access health services if this was the wish of the family and the patient. Where a person was considered to be at imminent risk of a serious deterioration in physical or mental health due to the methods employed by healers the researcher informed the local CPN and senior researchers and medical staff at Kintampo Health Research Centre. In some cases where people with mental illness were chained, treatment with psychotropic drugs appeared to improve the mental health of the patient sufficiently for the family to release the person.

## Results

### The limits of family care

**Figure 2:**
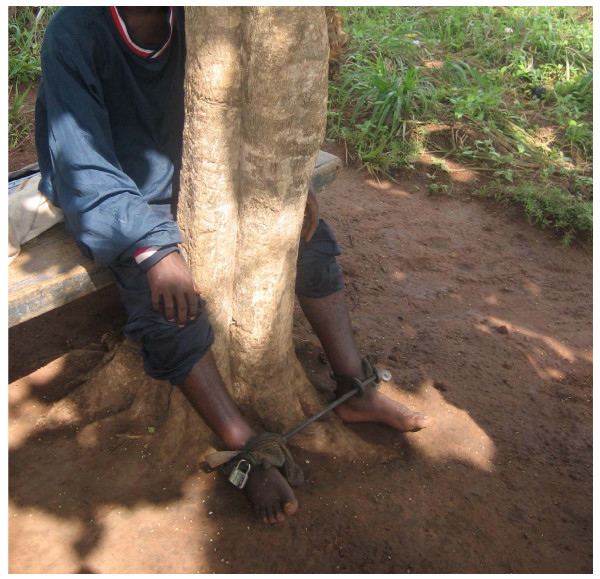
**Chains in use in a prayer camp**.

Almost all those with mental illness encountered in this research had been chained, either at home, or within healing centres. The most common form of restraint was metal shackles which enclosed the ankles and were attached to a tree or post (see figure 2). Occasionally people with mental illness were chained to logs.

Caring for a relative with mental illness placed enormous financial and emotional strain on families, many of whom were already living with limited resources. Carers described struggling to manage agitated and aggressive behaviour. Some reported that a son or daughter had made threats of violence. One woman for example, had been chained after threatening her grandmother with a knife. Another man was chained to a log to prevent him from preaching loudly during the night and attempting to stop speeding traffic. In a few cases, some informants reported being injured by their relative, such as one mother whose daughter had thrown a piece of metal at her which had cut her shin very deeply. For some informants, such behaviour led to the family chaining their relative in order to protect themselves.

Outside of the extended family and neighbours, there are few avenues of support for those in Kintampo districts attempting to care for a relative with mental illness. Agitated or aggressive behaviour often persuades the family to seek help at shrines, churches or hospitals, since they are no longer able to manage their relative at home. The churches and shrines present the most obvious and accessible resources to assist in restraint and management, compared to the long and expensive journey to the psychiatric hospitals on the coast, although almost all of those interviewed had also sought psychiatric treatment from the hospitals at some point during the course of the illness. However, given the poor quality of care within the psychiatric hospitals, the limited efficacy of psychotropic medication for some informants, as well as unpleasant side effects, many families saw little evidence of better alternatives within biomedical treatment. This father of a young man at the shrine, describes how he had tried both biomedical and Christian treatment to no effect:

*When the illness first occurred I took him to Ankaful *[psychiatric hospital] *for his brain to be examined*, [...] *They didn't explain anything, and prescribed some medicine to give him. They told us that when the medicine was finished we should go to Sunyani. So when the medicine was finished, we went back for more. Yet still, the illness was getting worse, so we went to a prayer camp*.

Interview with father of Kwasi, shrine, 18^th ^June 2008

Spiritual perspectives on mental illness reinforce the popularity of the shrines and churches, since, unlike the hospitals, they address factors such as evil spirits, sorcery and witchcraft, which are commonly seen to have caused mental illness.

With no ambulance service or medical staff available to provide an escort, families faced a challenging task bringing disturbed and agitated relatives to places of treatment, particularly if using public transport, for most the only affordable means. One relative described how her brother had to be restrained by seven men in order to bring him to the shrine for treatment. This family paid the police who used their handcuffs to restrain the man and bring him to the shrine in a car.

Chaining of patients is generally conducted with the co-operation of the families who bring their relatives to healing centres. Indeed, several family members reported purchasing the shackles used to restrain their relative. At least four families visited had also resorted to chaining their mentally ill relative at home. Carers interviewed at the shrine and churches were generally accepting of the need to chain their relative if he or she was 'aggressive', 'roaming around', disruptive or using cannabis. Being 'disturbing' (*gidigidi)*, and 'roaming' (*kyinkyin)*, were common reasons for the use of chains. The father of Kwasi viewed the use of chains as important to control his son when he became loud, hyper-talkative and disruptive, behaviour which we had witnessed on our visits:

*He was mostly chained to a tree. He was released whenever he calmed down. That is how I saw it....When the sickness came, he made a noise and they chained him to a tree*.

Interview with father of Kwasi, shrine, 18^th ^June 2008

Some parents also seemed haunted by a fear of their child becoming vagrant, a common fate for those with mental illness who often seemed compelled to wander far from home. Akua was living in a prayer camp and had had a severe mental illness for 10 years. She and her mother provide a typical description of this restlessness that could lead to people with mental illness wandering into the bush:

Akua: *I will be standing there talking with someone, and if I go out I could get lost. And if I get lost, I don't know where I am going. If someone calls me*...

Mother:*If it comes likes that she can't stay at home, it makes her go walking into the bush, it won't allow her to stay at home*.

Interview with Akua and mother, prayer camp, 8^th ^May 2008

Vagrants are a common sight in Kintampo town and at the roadside, most of whom showed signs of mental illness. For some the use of chains was a means of preventing this fate for a son or daughter and of keeping him or her within the family home. We were told moving stories of family members who had searched for their son or daughter for months; one man had had to go as far as Niger in search of his brother. During the course of fieldwork, one of the cases we had interviewed disappeared from home.

### Madness and the loss of social status

However, despite this desire to restrain and contain agitated, restless or potentially violent relatives, it was evident that chaining and other forms of harsh treatment such as beatings, were also embedded within concepts of mental illness which were influenced by spiritual and moral understandings of the person and society. Descriptions of the typical 'madman' provided by informants portrayed him as dirty, unkempt, anti-social, and beyond the norms of human behaviour. Madness is also commonly associated with dangerousness. The mad are unpredictable, irrational and potentially violent, as in this young man's description of a woman who had lived in his compound and become mentally ill:

...*it comes and goes. But when it comes and she sees... she sees you, she can just pick anything she see on the floor and throw it on you, and throw it to hit you, maybe to wound you or to kill you. She'll be sitting down, talking by heart, insulting people, don't you see? Then laughing....doing all sorts of things*.

FGD with young people, 30^th ^April 2008 in English

Such behaviour directly contravenes social ideals of personhood, in which taking responsibility for others, such as parenting children, is valued as the mark of adulthood [25]. The Ghanaian philosopher, Kwasi Wiredu, claims that for the Akan, 'a person in the true sense is not just any human being, but one who has attained the status of a responsible member of society', that is someone who 'is able to achieve a reasonable livelihood for himself and family while making non-trivial contributions to the well-being of appropriate members of his extended kinship circles and the wider community' [26]. All of those we met suffering from chronic mental illness were falling well outside this ideal since most were unable to work, and almost all were unmarried and childless. Given this failure to achieve these markers of adulthood and responsibility, the status of the mentally ill was in some way analogous to that of a child.

This loss of social status is captured by the concept of a 'spoiled' human being, which was used by some informants to describe those who had become mentally ill. Akua told us:

'They say that now I'm spoilt. I'm not a human being anymore.'

Interview with Akua, prayer camp, 8^th ^May 2008

The Twi s*ɛ*e, translated here as 'spoilt' is a polysemic word, used to describe moral corruption, bewitchment or bedevilment, rotten food, something gone bad or wasted. One of the pastors for example, explained how the devil had 'spoiled' a man through alcohol. A 'spoiled' status, as in Akua's statement, implies a loss of a person's essential humanity and carries a moral charge. The implication is that those with mental illness may be subject to forms of harsh treatment which would not be permitted to other categories of person.

### Chains as part of treatment

The use of chains and shackles formed a routine part of treatment in the shrine and churches visited. Every healer visited during the research, whether a Christian pastor or a traditional healer, employed shackles on those with mental illness. Patients were commonly chained when they were first admitted to a shrine or prayer camp and removed once the person became calmer, sometimes after a few days, or a couple of weeks. In very agitated cases, or where the person was thought to be likely to run away, the chains were kept on for months. A common concern for healers and carers was that young men who had been smoking cannabis would run away to smoke if they were not chained. Pastors and traditional healers in the area argue with some validity that they are providing a vital service for the management of those with mental disorders and many pleaded for greater recognition of their contribution. Their struggles to manage agitated and sometimes aggressive patients, as well as distressed and despairing relatives, called for resources which few were able to provide. None of the healers had any form of accommodation of a standard suitable to forcibly detain patients. Using shackles therefore enabled healers to enforce treatment such as herbal medicine, 'fasting' and praying. In the case of Christian pastors, the chains then became part of fulfilling their divine mission. One pastor argued that he could not afford to build accommodation at his prayer camp, so had no alternative but to use chains to carry out the work God had called him to:

As for me, it is something God has given me, so if I could take whoever comes here I would be pleased, but the financial problem. And when the mad people come first it is difficult, so we have to chain them to be able to pray for them for the evil spirit to leave them, for them to have their peace. There is no money, otherwise we wouldn't chain them, and I also don't have a room to put them in.'

Interview with Prophet Agyei, owner of prayer camp, 23^rd ^June 2006

The two other pastors running prayer camps similarly reported using chains to manage violent behaviour and protect others in the public space:

*So he comes and we get him to sit down, and we are going to pray, and you are about to pray for him and he will want to hurt you. Yes. So sometimes we put chains on their legs so that they won't hurt anybody. Some they go too 'high', so you have to put chains on their legs so the person becomes calm and you pray for him*.

Interview with Pastor Owusu, owner of prayer camp 14^th ^May 2008

*At the time they brought him, it was very difficult. He was very violent. When it happened like that, he got new strength. So we had to put him in chains because if you leave him, he could harm somebody. We had it tough before we were able to chain him*.

Interview with Maame Grace, owner of prayer camp, 16^th ^October 2008

However, shackling is not always a response to violent or uncontrolled behaviour. Madness was commonly seen by informants as punishment for transgressions and moral failings such as breaking of taboos, stealing and adultery. Attributions for the mental illness of some in this study included the use of sorcery and witchcraft, possession by evil spirits, and adultery. Madness is also associated with smoking cannabis which is strongly morally sanctioned, representing a form of marginalised and anti-social behaviour, particularly among young men. In line with this moral perspective on mental illness, chaining and beating were used for punishment and discipline as well as restraint within the prayer camps and shrines. Informants described how people were beaten with sticks, belts and strips of metal and rubber. At the shrine several informants described how patients were beaten if they refused to take the herbal medicine, or as punishment for running away. Since the status of those with mental illness was akin to an unsocialised child, beating mirrored common methods employed in the disciplining of children, such as beatings with sticks, although often to a more brutal degree than would be generally acceptable.

Beatings were also part of treatment to rid the person of evil spirits which were perceived by both pastors and traditional healers to lie behind much mental illness. Informants in this study described being beaten to drive away evil spirits such as *mmoatia *(small forest-dwelling spirits which were reported to possess several informants, causing madness), or to extract a confession of wrong-doing or witchcraft. A mother of a patient at the shrine described how her daughter had been beaten so severely at a prayer camp, that she had been left permanently scarred:

*There *[at a prayer camp] *they beat her severely with a belt, today you can see her back, all over her back. They said she should say she is a witch, but she is not a witch, and so they beat her severely with a belt, she had wounds all over her back*.

Interview with mother of Yaa, shrine, 25^th ^July 2008

Extracting a confession was viewed by healers as important since if the person failed to confess their wrong-doing, they could not be healed.

*There are people maybe they did something evil, and the evil they did brought the problem *[madness]. *There are people who after prayers they have to confess before the healing will come*.

Interview with Prophet Agyei, 23^rd ^June 2006

Healers argued that it was not people themselves who suffered from beatings, but the rather the bad spirits inside them. Hence beating was morally framed as part of the battle against the ultimate spiritual cause of mental illness.

In this view chaining and beating is seen as an essential part of the healing process. By contrast, the removal of the shackles serves a symbolic purpose for those who treat mental illness since it is tangible and dramatic demonstration of the efficacy of healing in effecting the transformation of the person from madness to health; from asociality to humanity. Two of the pastors interviewed had collected photographs of men and women who had attended their prayer camps where they were portrayed in a stereotypical state of madness, in chains with matted or 'bushy' hair, their semi-naked bodies partially covered by torn and dirty clothes. Maame Grace displayed 'before and after' shots side by side in an album, the 'after' photographs showing the person neatly dressed in new clothes, their hair cut or styled, released from chains. These photos echoed the story of the Gadarene madman healed by Jesus, which was cited by the pastors as a Biblical precedent for their work with the mentally ill. The photographs were therefore displayed, not as a shameful record of abuse, but as a visible demonstration of the efficacy of healing. This was a view shared by some of the carers and even people with mental illness, who saw the removal of chains as evidence of improvement.

### Voices of dissent

However there were those in Kintampo who disagreed with the harsh treatment given to people with mental illness by pastors and traditional healers. Some family members interviewed were unhappy with the use of chains on their relatives. Some had refused to use the prayer camps or shrines for this reason or had taken their relative away from such places. The mother of Alice, who suffered from a long-standing mental illness, had previously sent her to a shrine where she had been chained. She explicitly compared the treatment of her daughter to that of an animal, and claimed her daughter's right by contrast to be treated as a human being:

*Ei! It is worrying. It is very sad. She is not a dog that anybody can chain like that. If she gets up to go to the toilet you have to remove the chain so she can go. So the person looking after her feels very sad. It is something to make you sad*.

Interview with mother of Alice, Kintampo, 23^rd ^July 2008

Alice's mother's view is particularly striking when one considered how she had been treated by her daughter when she was unwell. Alice had frequently publicly insulted her mother, which the researchers had witnessed. This had progressed to a physical attack on her mother, however she had refused to punish her:

*When she threw the piece of metal, it hit me here *[pointing to shin]. *It cut me down to the bone... [...] Her brother said he would beat her, but I stopped him. This is because she wasn't in her own mind. If she was in her own mind, she wouldn't hit me with a piece of metal like that*.

Interview with mother of Alice, Kintampo, 23^rd ^July 2008

Importantly, in contrast to the viewpoint of people like Prophet Agyei, Alice's mother framed her daughter's behaviour as not being of her own volition, but rather 'out of her mind': *enyε n'adwene*, literally 'she did not have her mind'. This phrase carries not only the connotation of losing control of one's own thoughts and behaviour, but of not being one's true self.

It was striking how few of those who had been subject to the use of chains or beating complained of their treatment at the hands of the pastors and fetish priests. However some of those who had been chained were clearly very distressed by their treatment and expressed resentment towards the healer and the relative who had placed them in chains. It was noticeable that the strongest criticism was voiced by those who were most unwell. Their complaints were dismissed by carers and healers as symptomatic of the rebellious behaviour which was part of their madness and their lack of insight into their mad condition. On one visit to Maame Grace's prayer camp for example, a teacher who was shackled begged me to release her and expressed her anger towards 'that woman' the pastor, who she said had called her a witch. Another, Moses, angrily contested his treatment by his mother who had brought him to the shrine, and told us about the beatings he had received and the unpleasant sensations he experienced when taking high doses of herbal medicine which induced a semi-conscious state, and caused diarrhoea. Another male patient at the shrine complained of the degradation of sitting in his own urine whilst in chains and threatened to report the priest once he was released.

Once they were recovered many of those who had been chained or otherwise harshly treated, conformed to the general view that their treatment was justified on the grounds of their madness. Most informants who had recovered sufficiently to be interviewed expressed little resentment towards the healer who had chained them, viewing it as a necessary part of the process of healing and perhaps unavoidable given their disturbed behaviour. Some stated that the chains had 'helped' because it had made them comply with the treatment or had acted as a form of 'negative reinforcement':

*When I first came here, I was put in chains because they thought I would run away. I was in chains for three days and was given some herbal medicine to take. I don't consider this as maltreatment but a way to treat me and see to it that I am well*.

Interview with Kwabena, shrine, 18^th ^October 2008

Notably some young men had chosen to stay and serve the pastors who had formerly chained them, training as pastors themselves and doing other work such as farming, maintenance and running errands. In turn they too assisted in the chaining of other patients with mental illness. For some informants the church and the shrine provided important social and material support and a refuge from stigma in the home community. The pastors, for example, provided subsistence such as food, clothing and accommodation in return for farming on their land, offering one option for survival in a region where there are very high rates of youth unemployment, particularly for young men.

## Discussion

### Enhancing mental health care

The challenges of providing mental health care in accordance with international human rights standards as shown in Kintampo have been noted elsewhere in Africa. Alem reports the use of ropes and shackles to restrain people with mental disorders in homes and traditional healing centres in Ethiopia. He remarks that in Ethiopia the provision of care in 'modern and traditional institutions' is not in accordance with protection of human rights as defined by 'western culture', however he argues that given the scarcity of resources for mental health care 'these procedures have protected many patients from vagrancy, and from the danger of deterioration which could arise from lack of treatment.' [27]. In Nigeria, Eaton and Agomoh report that traditional healers and "prayer houses" employ herbal remedies, chaining, beating, cutting of the skin, acid burning or starvation ("fasting") in the treatment of the mentally ill and 'serve a purpose as a means of containment'. The scarcity and expense of psychiatric services impedes their use by many. This is coupled with a lack of knowledge and doubts about the effectiveness of medical treatment for mental illness which is seen as caused by 'spiritual attack' [28].

As shown in this study, given the lack of state welfare provision in many African countries, responsibility for the care of those with severe mental illness lies with the family, leading to a significant carer burden [29-33]. A study in Nigeria showed that caregiver burden was higher where the relative demonstrated psychotic symptoms and 'uncooperative behaviour' [32]. As in this research, a study in Ghana of family responses to mental illness found that the family provided the main source of support in both rural and urban areas, leading to financial burden, emotional strain and social stigma. This research also reported how families had struggled to manage difficult and sometimes violent behaviour by people with mental illness towards family members, such as beatings and setting fires. Churches and mosques were reported to be important sources of material help [33]. In countries where social structure and health care has been devastated by war, government resources for the treatment and care of those with mental illness are even more scarce. A recent Channel 4 documentary shown in the UK, for example, provided graphic coverage of the use of chains within Sierra Leone's sole psychiatric hospital (staffed by the country's only psychiatrist), and within the compounds of traditional healers. In this film, both the psychiatrist and healers defended the use of chains as necessary to prevent their patients running away from treatment [34].

The *Global Movement for Mental Health *has explicitly linked the scaling up of mental health services with the protection of the human rights of those with mental disorders, promoting the development of policies and legislation to both enhance the provision of mental health care, and to protect human rights. The scarcity of accessible and high quality mental health care undoubtedly contributes to the continued popularity of traditional healers and prayer camps, and to the high attrition from psychotropic treatment. However as this research shows, other factors such as the belief in spiritual influences on mental health and a scepticism towards the effectiveness of biomedicine for mental disorders also result in families seeking alternatives to psychiatric treatment. Psychiatric hospitals are notorious within Ghana as elsewhere in sub-Saharan Africa for being over-crowded and under-funded. Quality of care is compromised by the low numbers of qualified staff, the paucity of on-going staff training, and a lack of psychosocial treatment and rehabilitation [35]. There are reports of beatings and the use of medication as punishment [36]. If psychiatric services are to be seen by people with mental illness and their carers as a viable alternative or adjunct to other forms of treatment, then they must not only reach out to rural communities, but also provide the highest standard of care which promotes human rights and respects the viewpoint of the person being treated.

### Local struggles and human rights

In this research it was notable that in contrast to international outrage within scientific journals and the media of Western states, the widespread use of shackles and other forms of maltreatment towards those with mental illness in Kintampo and the surrounding communities provoked remarkably little protest within the communities studied. Whilst there have been some criticisms of the harsh treatment of people with mental illness by traditional and faith healers in national newspaper articles, reports [13,37-39], and NGO campaigns in Ghana, these have had little impact at the local level. The Commission on Human Rights and Administrative Justice (CHRAJ) has an office in Kintampo only a short walk from one of the churches where chains are routinely employed. CHRAJ is a national government funded organisation whose mandate is to promote, protect and enforce fundamental human rights and freedoms through providing mediation, advocacy and legal support. Yet there has been no move to sanction the practice of chaining and other forms of maltreatment employed either by individual families or by healers. In August 2007 officials at the Kintampo office of CHARJ reported that no one had petitioned them regarding the human rights of people with mental illness, hence they had not taken up the case. Local health practitioners were also aware of the practices of local healers within their district.

From conversations and observation it appeared that families rather than the state were judged to be responsible for the welfare of their relative if they chose to use such treatment. Yet, it is at the level of state intervention that solutions are most often proposed. In Ghana for example, the attention of national leaders in psychiatry has focused on legislation to outlaw practices such as chaining. A new mental health law has been drafted which overtly adopts a 'human rights based approach' reflecting current international guidelines as articulated by WHO [40], although it has not yet been passed. This bill explicitly prohibits abuse within healing facilities, including traditional healers and 'spiritual mental health facilities' [41]. However the capacity of this act to effect imminent change in the treatment of the mentally ill is cautioned by the fact that within Kintampo districts existing legislation which should in theory protect the rights of the mentally ill, is routinely breached with impunity. The Mental Health Decree (1972), which forms current mental health legislation, does not address the issue of restraint or maltreatment by relatives or healers, however it does provide for the police to remove to 'a place of safety' any person suspected of suffering from mental illness who 'has been, or is being, ill-treated, neglected or kept otherwise than under proper control' [42]. The Constitution of Ghana which aims to protect the rights of all citizens, states that no person who is restricted or detained should be subjected to 'cruel, inhuman or degrading treatment or punishment' and 'any other condition that detracts or is likely to detract from his dignity and worth as a human being.' (Clause 15(2)) [43].

Leaving aside the question as to whether the chaining of those with mental illness is perceived by those who employ it as 'cruel, degrading, or inhuman', or whether it is rather viewed as an unfortunate necessity, even as mundane, the failure of existing legislation to impact on the treatment of the mentally ill in rural communities such as Kintampo, raises important questions about the viability of further legislation to protect people with mental illness from human rights abuses. National legislation may echo the best of the international human rights discourse with its language of freedom and rights, however many of those whom it seeks to protect would struggle to understand it, if they were even aware of its existence. The weakness of much human rights legislation, as has been cautioned of civic education campaigns in Malawi, is that 'the starting point is not the actual concerns and aspirations of the people, their particular situations in life and experiences of abuse, but freedom, democracy, and human rights as universal and abstract values.' [44] Where, as in Ghana, there is little faith in the efficacy of state apparatus, and law enforcement agencies are both over-stretched and corrupt, protecting human rights often falls to families and healers rather than the state. As argued by Farmer and Gastineau, 'rights attributed on paper are of little value when the existing political and social structures do not afford all individuals the ability to enjoy these rights, let alone defend them.' [10].

### Morality and rights

A further caution concerns the focus on individual rights within the human rights discourse employed by international agencies such as WHO. The approach to rights enshrined within much human rights legislation is largely founded on European concepts of the person as a self-determining individual. By contrast, the actions of family members and healers observed in this study reflect a concern with the safety and moral integrity of the group, rather than the individual rights of the person with mental illness. This reflects Ghanaian ideals concerning the sociality of human beings and relationships of reciprocity and responsibility, and the sanctioning of overt individualism. Within Ghana, as Englund [45] describes for Malawi, human rights may be viewed as grounded within a moral rather than legal framework, one which draws on 'traditional' morality as articulated at the shrines, and increasingly on Christian moral codes. Gyekye writes that 'Within the framework of Akan social and humanistic ethics, what is morally good is that which promotes social welfare, solidarity, and harmony in human relationships'. By contrast, moral evil (*bone*) is 'that which is considered detrimental to the well-being of humanity and society' [46]. In this view rights carry responsibilities, and are earned, rather than innate. This moral approach which emphasises both rights and responsibilities, suggests the need to engage with all the players involved in the use of methods such as chaining to find a way forward. It has been argued that in contrast to the absolutist division within human rights discourse between victims and violators, there is a need for 'less self-righteous modes of relating that are also more attuned to moral complexity: listening, compromise and the creation of new solidarities and practices of co-existence based on recognition of an imperfect shared humanness.' [47]. This approach may open a way for dialogue which avoids alienating those perceived within the human rights discourse as 'violators', and recognizes their sometimes legitimate concerns, for example for the safety of the community.

However it should be cautioned that such a moral perspective on rights and responsibilities may also be used to justify the maltreatment of the mentally ill as this research has suggested. The morally suspect status of the mentally ill may be seen to threaten the cohesion and moral integrity of the group, thus they may be excluded from entitlement to the rights otherwise accorded to morally upright and socialised human beings. Their rights are likely to be subsumed to the needs of the group thereby sanctioning the use of whatever means necessary to control behaviour which threatens this cohesion. As this research shows, to fail to address the issue of the chaining of the mentally ill and other practices of restraint, is to ignore the significant cost for those chained and beaten: socially, physically and psychologically. Several patients had suffered lasting physical damage as a result of being chained for long periods, such as muscle wasting and shortening. Many bore scars on their ankles, evidence of the abrasion caused by the shackles. Some were resentful of the treatment received at the hands of their families, or of healers or pastors into whose care they had been entrusted by family members. In some cases this can lead to a breakdown in family relationships from which some families never recover. The ultimate risk surrounding attempts at chaining and restraining those who are agitated or aggressive, was tragically illustrated during the course of fieldwork. In May 2008 a young police officer experienced a mental breakdown whilst in a church in Kintampo, and began to behave aggressively, smashing objects and shouting. As yet the facts of the case have not been verified, however it appears that in the course of attempts to restrain him by church elders, the man's neck was broken and he died.

There are some signs in Kintampo of an uneasiness with the practice of chaining, and a desire for other alternatives on the part of those involved in treating the mentally ill. A few families strongly resisted the use of chains on their relatives and chose to forgo the treatment offered by spiritual healers where such methods were employed. A pastor whose church routinely chains the mentally ill to trees, expressed the opinion that such treatment is 'not the best', and stated his desire for funding so that better facilities for the confinement of patients could be provided. Since 2008 representatives from CHRAJ, the District Health Management Team, traditional and faith healers, carers and people with mental illness are co-operating with the MHaPP in Kintampo to promote the human rights of people with mental illness in the district. Initiatives such as these, which establish a dialogue with local actors, could begin to address the factors which contribute to the continued use of chaining and other forms of abuse, and work alongside families and healers to protect and promote the rights, dignity and health of those with mental illness.

### Limitations

This study suffers from a number of limitations most of which are inherent in the anthropological approach with its focus on 'ethnographies of the particular' [48] and the use of key informants. Whilst it provides an indepth study of factors surrounding responses to mental illness within the communities under study, caution should be exercised in generalising these findings elsewhere since the sample size is small and particular personal, historical, social and cultural factors will vary. Ideally a greater engagement between such qualitative anthropological studies and quantitative research utilising standardised instruments along the lines suggested by De Jong and Van Ommeren for cross-cultural epidemiology [49] could provide a means of counterbalancing the limitations of both methodologies within international mental health research. Whilst the long period spent in the field may have helped to minimise the effect of the researchers during participant observation and interviews to some degree, the presence of both educated Ghanaian researchers and a white European researcher undoubtedly influenced the responses provided in both positive and negative ways. For example, the informants may have been able to say things to a 'stranger' that they could not say to a member of the community, but equally they may not have been willing to disclose other facts to 'strangers'. The use of Twi as the lingua franca may have disadvantaged those for whom it was not their first language, and the process of translation inevitably leads to some loss or distortion of meaning. We attempted to minimise this through transcription first into Twi and through explanation of the Twi words used where these were polysemic and had no direct translation in English.

## Conclusion

There remains a gap between the global discourse on health (one conducted largely in English, the language of power), which is echoed within the corridors and conference rooms of ministries of health within Ghana and other low-income countries, and the conversations and decisions around health care which take place at community level. This research illustrates some of the challenges faced by families in supporting relatives with mental illness, and the suffering endured by those who are subjected to chaining, beating and other forms of harsh treatment within healing centres and family homes. As this study has shown, many families allow their relatives to be chained in order to provide treatment which is perceived to be in their best interests, and to control and punish difficult behaviours. Counter-intuitively, the use of chains can therefore represent an attempt to maintain the care of severely mentally ill relatives in the absence of avenues of support and at great emotional, social and material cost. Such practices are also rooted within accepted responses to mental illness within the study area, drawing on historical, cultural and symbolic meanings, and thus do not evoke the level of protest that might be expected within a discourse of human rights. Indeed, the emphasis on individual human rights employed by international agencies may fail to engage with local concerns underlying practices of restraint, and the need to provide viable alternatives which will support both those with mental illness and their families.

In arguing for a 'scaling up' of mental health care *The Global Movement for Mental Health *and WHO should be wary of a 'one size fits all' approach that may fail to recognise local resources and concepts of mental health and illness which sometimes sit uneasily with biomedical approaches to psychiatric treatment. As argued by Alem for Ethiopia [27], mental health care predicated solely on Western models is unlikely to be realistic in the context of the limited resources available in low-income countries of sub-Saharan Africa, nor may it be the best response to the particular needs of rural communities. Research in Malawi suggests that greater knowledge of biomedical models of mental illness may not necessarily reduce carer burden [50]. However providing an outreach service for people with schizophrenia in rural India which provided psychosocial support and advice, alongside psychotropic medication, was shown to reduce symptoms, disability and family burden [51]. In rural Nigeria other approaches to facilitating access to mental health services and working with families include involving family members in providing treatment and combating stigma [52], and the training of village health workers [28]. Given the important role of families and informal healers in Ghana in providing care and managing the challenging behaviours sometimes displayed by those with serious mental illness, mental health services need to consider how best to strengthen family resources and engage with local healers to present realistic alternatives to chaining. There is also a need to confront deep-rooted historical and cultural practices which inform responses to mental illness at the level of families and the broader society. This is evidently more difficult, since it involves the changing of attitudes. Legislation alone is unlikely to alter practices used for the restraint of those with mental illness, unless it is coupled with a commitment to funding mental health services. These services must be flexible enough to reach out to rural communities if they are to be accessible to families with few material or financial resources to access treatment at more distant health care facilities. They must also be creative enough to overcome the limitations of a strict biomedical psychiatry and find ways of working with local families and healers to improve the care of those with mental illness and relieve something of the burden felt by many carers.

Legislation to protect the human rights of people with mental illness is undoubtedly a vital tool to regulate abuses within both government and private treatment facilities. However such legislation is likely to prove harder to implement within small rural communities which are distant from the reach of the state, and will be beyond the means of many to exploit for their protection. Ultimately engaging with local actors as they struggle to live with mental illness and search for a cure, may promise more in terms of changing responses to mental illness than creating legal sanctions which are unlikely to provide immediate benefits in the short term.

## Appendix 1: Outline of methods

### Case studies

▪ Visits to case families at home and/or healing facility

▪ Participant observation of everyday life e.g. work, family interactions, social activities

▪ Observation and conversation with people with mental illness and family/friends

▪ Semi-structured interviews with people with mental illness and family members

### Healing resources for mental illness

▪ Participant observation of healing rituals and practices at shrines and prayer camps

▪ Visits and observation at health facilities (psychiatric hospitals, CPN clinics, general hospitals, rural clinics) and conversation with health workers

▪ Visits to mental health NGOs

▪ Semi-structured interviews with healers, pastors and people attending healing facilities

### Concepts of and attitudes towards mental illness

▪ Content analysis of media representations of mental illness e.g. newspaper articles, TV, films

▪ Exploration of popular knowledge of mental illness as revealed in proverbs, folk tales, symbolic representations etc.

▪ Focus groups with nurses, church members, Muslims, young people, parents etc.

## Competing interests

The authors declare that they have no competing interests.

## Authors' contributions

UMR conceived of the study, and developed its design. She participated in all aspects of the research and prepared the draft of the manuscript. SN completed the interviews and focus group discussions, participated in fieldwork, and helped develop the research questions. SN and EA read the manuscript and provided additional comments. All authors edited and approved the final manuscript.
